# Resonance modes in microstructured photonic waveguides: efficient and accurate computation based on AAA rational approximation

**DOI:** 10.1515/nanoph-2024-0755

**Published:** 2025-03-18

**Authors:** Felix Binkowski, Fridtjof Betz, Martin Hammerschmidt, Lin Zschiedrich, Sven Burger

**Affiliations:** Zuse Institute Berlin, 14195 Berlin, Germany; JCMwave GmbH, 14050 Berlin, Germany

**Keywords:** photonic crystal fiber, fundamental modes, AAA rational approximation, nanostructured waveguide, leaky modes

## Abstract

We present a framework for the efficient and accurate computation of resonance modes in photonic waveguides. The framework is based on AAA rational approximation with the application of special light sources. It allows one to calculate only relevant modes, such as the fundamental resonance modes localized in the central core of the waveguides. We demonstrate the framework using an example from the literature, a hollow-core photonic crystal fiber. This waveguide supports many other modes, such as cladding modes and higher-order modes. These nonrelevant modes are not calculated, so that challenging post-processing with mode filtering is not required.

## 1 Introduction

Resonance effects localize optical fields in dielectric fibers and other waveguides and allow for well-defined propagation of light over large distances. Application areas include communication technology [1], nonlinear optics [2], sensing [3], and imaging [4]. Resonances are the solutions to the source-free Maxwell’s equations with open boundary conditions and they are given by electromagnetic fields, the so-called resonance modes, with complex-valued eigenvalues [5]. Numerical methods are used to compute the resonances [6], [7], where often many modes of different types are calculated within a dense spectrum.

The functionality of photonic devices based on waveguides is driven by the so-called fundamental resonance modes. These modes are characterized by a localization of the electromagnetic field energy in the central core of the waveguides, which enables low-loss guidance of the light. Furthermore, microstructuring [8], [9] of the waveguides leads to modes that are localized in the cladding of the systems [10], and fibers with a hollow core support higher-order modes [11]. Such types of modes are often nonrelevant and make the calculation of the fundamental resonance modes a challenge, as a large number of modes must be calculated and the fundamental resonance modes must then be selected by post-processing. Another challenge is that the eigenvalues of the fundamental resonance modes can have extremely small imaginary parts compared to the real parts due to the low losses [12], [13]. This leads to very high demands on the numerical accuracy. Therefore, there is a need for approaches that can calculate the fundamental resonance modes of photonic waveguides efficiently and accurately.

Rational approximation is an effective approach for investigating resonant photonic systems. The resulting approximations give the poles and other key figures of the corresponding photonic response functions. The AAA algorithm [14] is a powerful tool for rational approximation. It can be used for the approximation of nonlinear eigenproblems, where the resulting rational eigenproblems can then be solved with suitable numerical methods [15], [16], [17]. Recently, approaches have been presented, where rational approximation with [18], [19] and without [20] applying the AAA algorithm is used to directly solve eigenproblems, i.e., to compute the resonance modes associated to the poles of the response functions of interest.

In this work, we present a framework based on AAA rational approximation to compute the fundamental resonance modes of photonic waveguides. The modes are calculated with the approach proposed in Ref. [19] using special light sources. We apply the framework to a hollow-core photonic crystal fiber (HC-PCF), where the microstructuring of the fiber leads to the existence of many cladding modes and the hollow core enables the presence of higher-order modes. The framework allows for an efficient and accurate computation of the fundamental resonance mode, where the other types of modes are not calculated. Challenging post-processing is therefore not necessary. The results are compared with the results obtained by the Arnoldi algorithm, which is a standard tool in the field of computational photonics.

**Figure 1:**
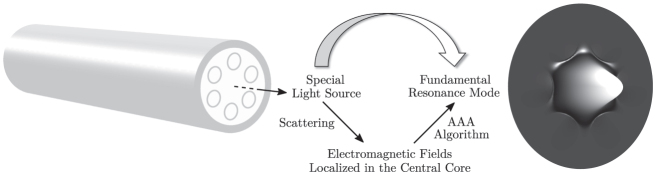
Computation of the fundamental resonance mode of a microstructured waveguide. The waveguide is illuminated with a light source located at the center of the system. The resulting scattered electromagnetic fields are superposed based on AAA rational approximation. The light source is chosen so that it couples mainly with the fundamental resonance mode and not with other modes. This special choice of the source enables an efficient and accurate computation of the fundamental resonance mode of the system.

Figure 1 outlines an application example of the framework. The fundamental resonance mode of a photonic waveguide is characterized by a localization of the corresponding electromagnetic field energy in the central core of the waveguide. A specially selected light source, which is located at the center of the system, has a significant coupling with the mode. Application of the AAA algorithm to the fields caused by the source yields the fundamental resonance mode.

## 2 Computing resonance modes with the AAA algorithm

The AAA algorithm [14] gives an approximation of a scalar-valued function *f*(*z*) by a rational function *r*(*z*) in a barycentric representation. A number *M* of freely selectable sampling points $z_{k}\in Z\subseteq C$ and corresponding function values *f*_*k*_ = *f*(*z*_*k*_) are the input for the algorithm. The algorithm greedily adds sampling points ${\hat{z}}_{j}$ to a subset $\hat{Z}\subset Z$ , together with the corresponding function values ${\hat{f}}_{j}$ . Then, each iteration within the algorithm leads to a rational approximation *r*(*z*) of order *m* − 1,

(1) $$r\left(z\right)=\frac{n\left(z\right)}{d\left(z\right)}=\left.\overset{m}{\underset{j=1}{\sum }}\frac{{\hat{w}}_{j}{\hat{f}}_{j}}{z-{\hat{z}}_{j}}/\overset{m}{\underset{j=1}{\sum }}\frac{{\hat{w}}_{j}}{z-{\hat{z}}_{j}}\right.,$$

where the weights ${\hat{w}}_{j}$ minimize the error

(2) $$\underset{z_{k}\in Z\backslash \hat{Z}}{\sum }|f_{k}\;d\left(z_{k}\right)-n\left(z_{k}\right){|}^{2}.$$

The least squares problem in Eq. (2) is solved using a singular value decomposition with the constraint $\sum_{j=1}^{m}|{\hat{w}}_{j}{|}^{2}=1$ . The solution of the least squares problem requires *m* ≤ *M*/2. The AAA algorithm also directly provides the underlying key figures of the rational approximation, such as the poles $z_{n}^{\text{pole}}\in C$ and the residues $a_{n}\in C$ .

In the following, we consider a physical vector-valued quantity $f\left(z\right)\in C^{N}$ which is the solution of the linear system of equations

(3) A(z)f(z)=s(z),

where $A\left(z\right)\in C^{N\times N}$ is the system matrix and $s\left(z\right)\in C^{N}$ is an imposed source term. It is further given that $f_{k}=L\left(f_{k}\right)$ , where $L:C^{N}\to C$ is a linear mapping and *f*_*k*_ are the function values of the scalar-valued function *f*(*z*) as introduced above. Then, the AAA algorithm also yields the vector-valued approximation

$$r\left(z\right)=\left.\overset{m}{\underset{j=1}{\sum }}\frac{{\hat{w}}_{j}{\hat{f}}_{j}}{z-{\hat{z}}_{j}}/d\left(z\right)\right.\approx f\left(z\right)$$

and the corresponding vector-valued residue

(4) $$a_{n}=\overset{m}{\underset{j=1}{\sum }}\left[\frac{{\hat{w}}_{j}}{z_{n}^{\text{pole}}-{\hat{z}}_{j}}/\frac{\partial d}{\partial z}\left(z_{n}^{\text{pole}}\right)\right]{\hat{f}}_{j},$$

where the vectors ${\hat{f}}_{j}$ are defined by the relation ${\hat{f}}_{j}=L\left({\hat{f}}_{j}\right)$ . Note that the weights ${\hat{w}}_{j}$ and poles $z_{n}^{\text{pole}}$ in Eq. (4) are the same as those used for the scalar-valued rational approximation *r*(*z*) from Eq. (1).

When a pole $z_{n}^{\text{pole}}$ has a significant influence on the rational approximation *r*(*z*), we assume that **a**_*n*_ and $z_{n}^{\text{pole}}$ are a good approximation to an eigenpair of the corresponding nonlinear eigenproblem, i.e., $A\left(z_{n}^{\text{pole}}\right)a_{n}\approx 0$ . This means that the resonance mode **a**_*n*_ corresponding to the eigenvalue $z_{n}^{\text{pole}}$ has a significant coupling with the source term **s**(*z*) [19].

Note that, to compute the solution **f**(*z*) of Eq. (3), any black-box solver can be used. For the proposed approach, only access to the solution is required, i.e., the way in which the solution is calculated is not taken into account.

## 3 Application

**Figure 2:**
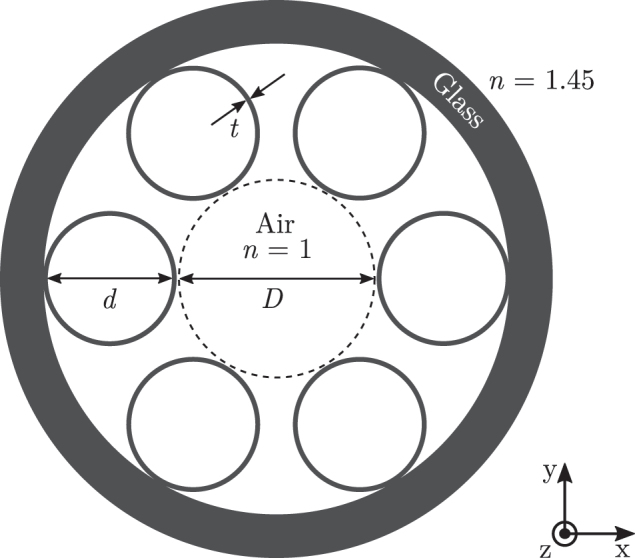
Sketch of the HC-PCF presented in Ref. [11]. The diameter of the central hollow core is *D* = 30 μm. The core is encircled by six nontouching glass rings with a wall thickness of *t* = *D* × 0.01. The inner diameter of the glass rings is *d* = *D* × 0.68. The HC-PCF is coated with thick-walled glass. The vacuum wavelength is set to *λ*_0_ = 1,500 nm.

We apply the approach presented to compute the fundamental resonance mode of a photonic waveguide from the literature, the HC-PCF introduced in Ref. [11]. The system is sketched in Figure 2. The longitudinal axis of the system is along the *z*-direction, and this dimension is much larger than the diameter of the cross-section of the system. Based on this, we model the HC-PCF with an infinite length in the *z*-direction and we assume a harmonic dependence of the scattered electric fields $E\left(x,y,z\right)\in C^{3}$ and imposed current densities $J\left(x,y,z\right)\in C^{3}$ on the *z*-coordinate, i.e., $E\left(x,y,z\right)=E\left(x,y\right){\text{e}}^{\text{i}k_{z}z}$ and $J\left(x,y,z\right)=J\left(x,y\right){\text{e}}^{\text{i}k_{z}z}$ , where $k_{z}\in C$ is the propagation constant. With this, in the steady-state regime, light scattering in the system can be described by the time-harmonic Maxwell’s equation in second-order form,

(5) $$\begin{array}{c} \nabla_{k_{z}}\times \mu^{-1}\nabla_{k_{z}}\times E\left(x,y\right)-\omega_{0}^{2}\epsilon E\left(x,y\right)=i\omega_{0}J\left(x,y\right), \end{array}$$

where $\nabla_{k_{z}}={\left(\partial_{x},\partial_{y},ik_{z}\right)}^{T}$ . The material is characterized by the complex-valued permittivity and permeability tensors *ϵ*(*x*, *y*) and *μ*(*x*, *y*), respectively. The angular frequency *ω*_0_ = 2*πc*/*λ*_0_ is a fixed parameter, where *λ*_0_ is the vacuum wavelength.

The electric field in the HC-PCF is excited with a singular electric current density on a line along the *z*-direction,

$$J\left(x,y\right)=j\delta \left(\left(x,y\right)-\left(x_{0},y_{0}\right)\right){\text{e}}^{-\text{i}k_{z}z_{0}},$$

where (*x*_0_, *y*_0_, *z*_0_) is the position of the line source, *δ* is the Dirac delta distribution, and **j** is a constant strength vector. Since the fundamental resonance mode is localized in the central hollow core of the HC-PCF, we place the line source at the center of the system. We further consider an *x*-polarized line source, i.e., **j** = |**j**| × (1,0,0)^*T*^.

In order to solve Eq. (5), we use the solver JCMsuite, which is based on the finite element method (FEM). The thick-walled glass cladding of the HC-PCF is modeled to extend to infinity, i.e., open boundaries realized by perfectly matched layers are applied. We further exploit the double mirror symmetry of the system in the numerical implementations. Numerical convergence with respect to the FEM parameters is ensured. The corresponding settings can be found in the data publication [21].

### 3.1 Reference solutions from the Arnoldi algorithm

Reference solutions are obtained by applying the Arnoldi algorithm [6], [7], [22] within JCMsuite to the eigenproblem

(6) $$\begin{array}{c} \nabla_{k_{z,n}}\times \mu^{-1}\nabla_{k_{z,n}}\times E_{n}\left(x,y\right)-\omega_{0}^{2}\epsilon E_{n}\left(x,y\right)=0, \end{array}$$

i.e., the source-free form of Eq. (5). The Arnoldi algorithm requires a guess value for the eigenvalues *k*_*z*,*n*_, where *ω*_0_ is fixed. Then, it iteratively calculates a selected number of eigenvalues closest to the guess value, together with the corresponding resonance modes **E**_*n*_. The boundary conditions on the symmetry axes are chosen such that the polarization of the modes matches the polarization of the line source used for the AAA algorithm. In the following, for a traditional notation, the eigenvalues *k*_*z*,*n*_ are given in the form of effective refractive indices $n_{n}^{\text{eff}}=k_{z,n}/k_{0}$ , where *k*_0_ = 2*π*/*λ*_0_.

### 3.2 Rational approximation and eigenvalues

The optical response resulting from the illumination of the HC-PCF is investigated by the quantity $y^{T}E_{x}\in C$ , where $y\in R^{m}$ is a random vector [15] with a uniform distribution in the interval (−1, 1) and $E_{x}\in C^{m}$ are the *x*-components of the electric field **E**, which is determined on an equidistantly spaced cartesian grid in one of the four mirror symmetry planes. The circular computational domain with 100 points in the *x*- and the *y*-direction leads to *m* = 7,787 evaluation points. The electric field **E** is obtained by solving the scattering problem given by Eq. (5) at chosen sampling points $n_{j}^{\text{eff}}$ .

**Figure 3:**
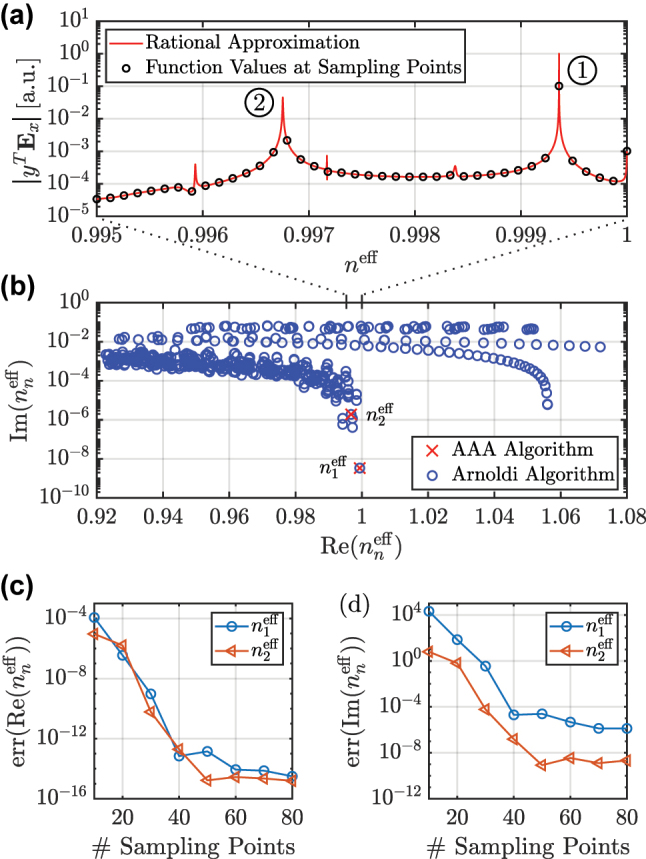
Illumination of the HC-PCF sketched in Figure 2 with an *x*-polarized line source located at the center of the system. Application of the AAA algorithm to the quantity $y^{T}E_{x}\in C$ , where $y\in R^{m}$ is a random vector and $E_{x}\in C^{m}$ are the *x*-components of the electric field **E**, which is evaluated at *m* spatial points in the computational domain. (a) Absolute values of the rational approximation based on 40 equidistantly spaced sampling points $n_{j}^{\text{eff}}\in \left[0.995,1\right]$ . The absolute values of the function values at the 40 sampling points are also shown. (b) Eigenvalues $n_{1}^{\text{eff}}$ and $n_{2}^{\text{eff}}$ resulting from the rational approximation and reference eigenvalues computed by the Arnoldi algorithm. (c) Relative errors $err\left(Re\left(n_{n}^{\text{eff}}\right)\right)=|\left(Re\left(n_{n}^{\text{eff}}\right)-Re\left(n_{n,ref}^{\text{eff}}\right)\right)/Re\left(n_{n,ref}^{\text{eff}}\right)|$ over number of equidistantly spaced sampling points in the interval *n* ^eff^ ∈ [0.995, 1], where the reference solutions $n_{n,ref}^{\text{eff}}$ are computed by the Arnoldi algorithm. (d) Relative errors of the imaginary parts of the eigenvalues.

We apply the AAA algorithm to *y* ^*T*^ **E**_*x*_ computed at 40 equidistantly spaced sampling points $n_{j}^{\text{eff}}\in \left[0.995,1\right]$ . The absolute values of the resulting rational approximation and of the underlying function values are shown in Figure 3(a). In Figure 3(b), the eigenvalues of the system are presented. We show 512 eigenvalues computed by the Arnoldi algorithm as reference solutions, where a guess eigenvalue of 0.9975 is chosen. The eigenvalues correspond to cladding modes, higher-order modes, and also to the fundamental resonance mode. In contrast, the application of the AAA algorithm with the special line source only yields relevant eigenvalues. They belong to the rational approximation shown in Figure 3(a) and they are selected based on the two significant peaks, marked with (1) and (2). These eigenvalues are given by $n_{1}^{\text{eff}}=0.9993596784939+0.000000003376i$ and $n_{2}^{\text{eff}}=0.996754264645+0.00000190093i$ . The eigenvalue $n_{1}^{\text{eff}}$ has the smallest imaginary part of all eigenvalues in the chosen range for the effective refractive index. The other eigenvalues [19] corresponding to the rational approximation, which arise due to the other peaks, the background continuum, or the eigenvalues outside the chosen range of the effective refractive index, and further details on the settings for the algorithm can be found in the data publication [21].

Figure 3(c) shows the relative errors of the real parts of the eigenvalues $n_{1}^{\text{eff}}$ and $n_{2}^{\text{eff}}$ over the number of sampling points for the AAA algorithm. For both eigenvalues, convergence up to errors smaller than 10^−14^ can be observed. Figure 3(d) shows the relative errors of the imaginary parts, where errors smaller than 10^−5^ and smaller than 10^−8^ for $n_{1}^{\text{eff}}$ and $n_{2}^{\text{eff}}$ , respectively, are achieved. The limitation of the accuracy can be attributed to the accuracy of the FEM scattering solver.

### 3.3 Fundamental resonance mode

To compute the fundamental resonance mode of the HC-PCF, we apply Eq. (4) using the weights, sampling points, and eigenvalues corresponding to the rational approximation from Figure 3(a). The matrix **A**(*z*) from Eq. (3) is the FEM system matrix, the vector **s**(*z*) corresponds to the imposed line source, and the vector **f**(*z*) is the scattered electric field in a finite-dimensional FEM basis. This means that, for the vectors ${\hat{f}}_{j}$ in Eq. (4), the FEM coefficient vectors corresponding to the electric fields **E** are used.

**Figure 4:**
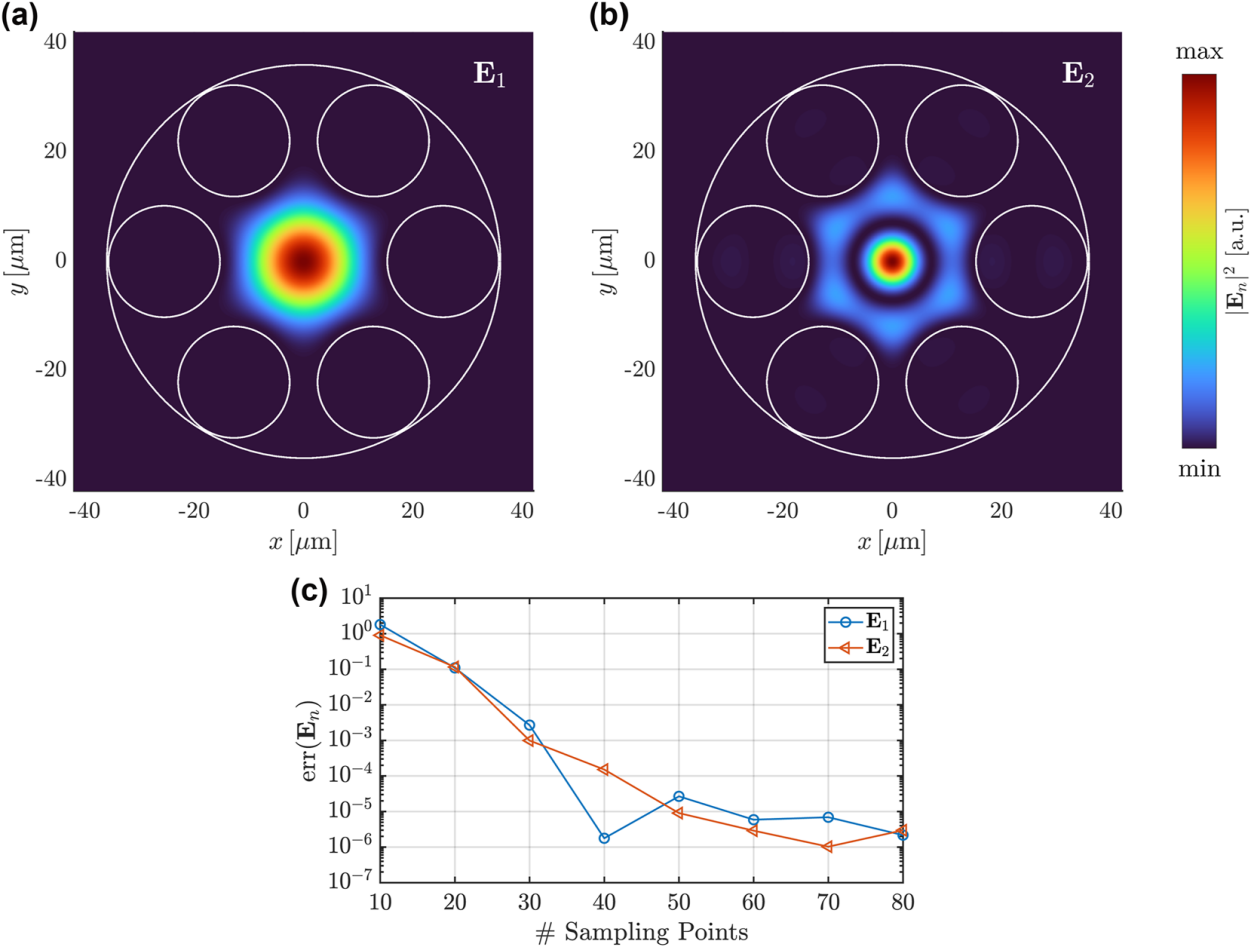
Computation of resonance modes of the HC-PCF sketched in Figure 2 using the AAA algorithm. (a) Electric field intensity of the fundamental resonance mode **E**_1_ corresponding to the eigenvalue $n_{1}^{\text{eff}}$ . (b) Electric field intensity of the resonance mode **E**_2_ corresponding to the eigenvalue $n_{2}^{\text{eff}}$ . The modes are scaled differently. (c) Relative error $err\left(E_{n}\right)=\left\|E_{n}-E_{n,ref}\right\|/\left\|E_{n,ref}\right\|$ over number of equidistantly spaced sampling points in the interval *n* ^eff^ ∈ [0.995, 1] for the AAA algorithm, where the reference solutions **E**_*n*,ref_ are computed by the Arnoldi algorithm. The norm ‖⋅‖ is defined as the square root of the electric field energy in the computational domain. The resonance modes **E**_*n*_ and **E**_*n*,ref_ are normalized such that their *x*-components at the center of the system are equal.

Figure 4(a) and (b) show the electric field intensities of the resonance modes **E**_1_ and **E**_2_ corresponding to $n_{1}^{\text{eff}}$ and $n_{2}^{\text{eff}}$ , respectively. We identify **E**_1_ as the fundamental resonance mode, as it has no nodal lines within the field pattern. Both modes are localized in the central hollow core of the HC-PCF. The chosen line source for the illumination of the system is located at the center and therefore exhibits a significant coupling with the modes. Figure 4(c) shows the relative errors of the two modes over the number of sampling points for the AAA algorithm. Convergence up to errors smaller than 10^−5^ can be observed.

Note that, in this work, we choose real-valued sampling points. The accuracy of the resonance modes and their eigenvalues could be further improved if sampling points in the complex plane are chosen. Further information on this topic can be found, e.g., in Refs. [18], [19], where adaptive sampling schemes are applied.

We further point out that other types of sources could also be used for the framework presented, e.g., a combination of multiple line sources, the field distribution of the fundamental resonance mode of a single-mode fiber, or a Gaussian beam. In this way, other types of modes than the fundamental resonance mode could be prioritized in the computation. For example, higher-order modes could be calculated by placing multiple line sources at positions based on the symmetry of the higher-order modes. Cladding modes could be calculated by placing line sources directly in the part of the fiber cladding of interest.

### 3.4 Computational performance

The calculation of eigenvalues and resonance modes using a framework based on AAA rational approximation differs in several aspects from standard approaches applied in computational photonics, such as the Arnoldi algorithm [6]. The AAA-based framework presented in this work essentially relies on solving various, independent scattering problems for obtaining a rational approximation of response functions, which then allows one to deduce eigenvalues and resonance modes. Advantages of this framework include the following aspects: (i) For systems with material dispersion, a linearization of the resulting nonlinear eigenproblem is not necessary. When using the Arnoldi algorithm, such a linearization must be implemented, which increases the degrees of freedom of the numerical realization [6]. (ii) Solving scattering problems enables the incorporation of sensitivities based on algorithmic differentiation [19], [23]. This means that also the sensitivities of the eigenvalues with respect to the system parameters are available with negligible computational costs. (iii) Domain decomposition algorithms can be applied in a straightforward way, which allows one to handle systems with multiple scattering [24]. (iv) The solution of a scattering problem within the AAA algorithm can be accelerated by using a preconditioner based on the computation of a solution at a neighboring sampling point. (v) If several computing nodes are available, the calculations at the sampling points can be parallelized. (vi) The use of problem-specific sources makes it possible to compute specific solutions only, as shown in this work. In contrast, other methods often rely on computing a larger spectrum and applying mode filtering a-posteriori, which implies high computational costs. A detailed and fair quantitative performance benchmark of the framework presented and standard approaches should investigate various examples, which enable to quantify the impact of the various aspects. Such a benchmark is beyond the scope of this work.

## 4 Conclusions

We presented a framework based on AAA rational approximation to compute the fundamental resonance modes of photonic waveguides. The framework was applied to an example from the literature, an HC-PCF supporting many cladding and higher-order modes. The scattering solutions at the sampling points for the AAA algorithm were superposed, reusing the poles and weights belonging to a corresponding scalar-valued rational approximation. The coupling of the underlying light source with the fundamental resonance mode of the HC-PCF enables an efficient and accurate computation of the mode.

The results were compared with the eigenpairs obtained using the Arnoldi algorithm. The Arnoldi algorithm solves the eigenproblem directly, i.e., without a source term, and it calculates all resonance modes together with the associated eigenvalues closest to a guess eigenvalue. The AAA algorithm is based on solving scattering problems at chosen sampling points, i.e., with using a source term, and, therefore, only calculates the modes that have a significant coupling with the applied source.

The framework presented is beneficial when the system of interest supports modes that are much more relevant than other modes, such as in the case studied in this work. The fundamental resonance mode of the HC-PCF investigated has an intensity maximum at the center of the system and it can be efficiently excited by a singular line source located at the center. Many of the additional resonance modes of the HC-PCF are modes that are localized in the cladding of the fiber or higher-order modes. They have an insignificant coupling with the line source, i.e., they are not calculated when using the AAA algorithm. This means that challenging post-processing with mode filtering, as may be necessary with the Arnoldi algorithm, can be avoided.

The computation of resonance modes based on the AAA algorithm is possible for any resonant system [25]. For example, in Ref. [18], a AAA-based approach is applied to an electromagnetic and an acoustic problem class and, in Ref. [19], a chiral photonic metasurface is numerically investigated using the AAA algorithm.
